# Supplementary material for: Epigenome-wide association study of circulating interleukin-6 connects DNA methylation to immunometabolic and inflammatory health
Source: Commun Biol. 2026 Feb 4;9:242. doi: 10.1038/s42003-026-09520-2 (PMC12905258; doi:10.1038/s42003-026-09520-2)
Supplement: Supplementary file 2 — Supplementary Information [file 42003_2026_9520_MOESM2_ESM.pdf]

Supplementary Information

Cohort Descriptions

Leiden Longevity Study (LLS)

The LLS[1] is a longitudinal, population-based cohort established in 2002, with the aim of investigating the genetic component of exceptional survival and its interaction with environmental factors. To this end, from 2002 until 2006, long-lived, Dutch, Caucasian siblings (n=944) were recruited with their offspring (n=1,671) and their offspring's partners (n=744).

Family eligibility required at least two long-lived living siblings who met a stringent, sex-specific age criterion (aged at least 89 years for males and 91 years for females). At the time of the study's initiation, less than 0.5% of the Dutch population fulfilled this requirement as an individual, and sibships with multiple eligible members were estimated to represent less than 0.1%[2].

Within the LLS population, offspring and other first-degree relatives are enriched for familial influences on longevity[1]. Their partners serve as controls, having comparable age, socio-economic status, location, lifestyle, and environmental factors but without this genetic advantage. Recruited, living subjects completed a pedigree, questionnaires, and a non-fasted venous blood sample was drawn for isolation of DNA, RNA, serum, and plasma.

Written informed consent for DNA collection and its use for genetic analyses was obtained from all participants prior to their enrolment into the study. Good clinical practice guidelines were maintained, and the study protocol was approved by the local Medical Ethical Committee of the Leiden University Medical Center. The LLS was supported by a grant from the Innovation-Oriented Research Program on Genomics (SenterNovem IGE01014 and IGE05007), the Centre for Medical Systems Biology, and the National Institute for Healthy Ageing (Grant 05040202 and 05060810), in the framework of the Netherlands Genomics Initiative / Netherlands Organization for Scientific Research.

Kooperative Gesundheitsforschung in der Region Augsburg (KORA)

The KORA study[3], established in 1984, is a series of independent population-based epidemiological surveys and studies of individuals living in the City of Augsburg, Southern Germany, and the 16 surrounding towns and villages. The KORA S4/F4/FF4 study[4] followed up this cohort with the aim of describing the prevalence, trajectories, and consequences of diabetes and other non-communicable diseases over 14 years. Specifically, the KORA S4 survey recruited members of the general population between 1999 and 2001 (n=4,281) and the KORA F4 study[5], which is used in this research, followed up a subset of these individuals aged from 32 to 81 years old (n=3,080) between 2006 and 2008.

In accordance with the Declaration of Helsinki, written informed consent was obtained from all participants prior to their enrolment into the study. Good clinical practice guidelines were maintained, and the study protocol was approved by the Ethics Committee of the Bavarian Medical Association.

Netherlands Twin Register (NTR)

The Netherlands Twin Register (NTR)[6-8] was established in 1987 to study genetic and environmental influences on phenotypic differences between individuals. To this end, data from twins and their families (currently more than 200,000 participants) from all over the Netherlands were collected, with a focus on physical and mental health, lifestyle, personality, cognition, and aging. In NTR Biobank, samples for DNA, RNA, cell lines, and biomarker assessment have been collected[4]. DNA methylation profiles, haemostatic profiles, and white blood cell counts were measured in the in samples obtained during the same blood draw session (hence all measures were obtained at the same timepoint).

Informed consent was obtained from all participants. The study was approved by the Central Ethics Committee on Research Involving Human Subjects of the VU University Medical Centre, Amsterdam, an Institutional Review Board certified by the U.S. Office of Human Research Protections (IRB number IRB00002991 under Federal-wide Assurance- FWA00017598; IRB/institute codes, NTR 03-180).

Cohort Measurements & Methods

Leiden Longevity Study (LLS)

Inflammatory markers Trained research nurses collected non-fasted whole blood from LLS participants in heparinized tubes and from the resulting plasma, IL-6 levels were measured using the PeliKine Compact Human IL-6 ELISA kit (Sanquin Reagents, Amsterdam, the Netherlands). The interassay cubic variance (CV), influenced also by dilutions for high cytokine values, was below 10%. Plasma C-reactive protein (CRP) was assayed with an automated enzymatic colorimetric method using a Modular P analyzer (Roche, Almere, The Netherlands) and CV was below 5%.

DNA methylation data DNA methylation data of whole blood samples was generated from 821 unrelated LLS participants by the Human Genotyping facility (HuGe-F, Erasmus MC, Rotterdam, The Netherlands) within the Biobank-Based Integrative Omics Studies (BIOS) consortium, funded by BBMRI-NL, a research infrastructure financed by the Dutch government (NOW 184.021.007)[7]. Following isolation according to standard procedures, genomic DNA (500ng) was bisulfite converted using the Zymo EZ-96 DNA methylation kit (Zymo Research Corp, Irvine, CA, USA). 4 µl was then hybridized on the Infinium HumanMethylation450 BeadChip array (Illumina Inc, San Diego, CA, USA) according to the manufacturer's protocol, which measures methylation at over 450,000 CpG dinucleotides across the epigenome.

The original IDAT files were generated by the Illumina iScan BeadChip scanner, which measures signal intensities in red and green colour channels. These were then imported into R for quality control and preprocessing. Data quality was assessed using both sample dependent and sample independent quality metrics reported by the Bioconductor package *MethylAid*[9] with default settings. This package investigates methylated and unmethylated signal intensities, as well as using control probes present on the array to monitor the efficiency of each step in the protocol.

Unreliable or outlying values were removed, including those indistinguishable from background noise (detection p-value > 0.01), based on a low number of beads (n<3), or with zero values for signal intensity. For each CpG site, methylation proportion was calculated from the extracted raw methylated (M) and unmethylated (U) probe intensities and a probe alpha (α) of 100. The

resulting beta values range from 0, which represented a completely unmethylated state, to 1, which represents a completely methylated state.

Finally, following background correction and probe-type normalization, the data was checked for outlying samples using plots of the first two PCs and any samples or probes with less than 95% success rate were removed. However, there were no outliers found in these checks indicating high quality data.

Model specification At each of i CpGs for j individuals (n=668), linear models for natural log-transformed IL-6 (in pg/mL) were fitted in R using the `lmFit` function from the Bioconductor `limma` package (see Formula 1).

$$\mathrm{DNAm}_{\beta_j} = \beta_0 + \beta_1 \cdot \ln(IL6)_i + \beta_2 \cdot \mathrm{age}_i + \beta_3 \cdot \mathrm{sexFemale}_i + \beta_4 \cdot \mathrm{CD8T}_i + \beta_5 \cdot \mathrm{CD4T}_i + \beta_6 \cdot \mathrm{NK}_i + \beta_7 \cdot \mathrm{Mono}_i + \beta_8 \cdot \mathrm{plate1}_i + \cdots + \beta_{17} \cdot \mathrm{plate10}_i + \beta_{18} \cdot \mathrm{arrayRow}_i \quad [1]$$

Age (in years) and sex (coded as 0 for males and 1 for females) were added as continuous and binary fixed effects, respectively. Cell type proportions were predicted from DNA methylation data using the IDOL algorithm[10]. Technical variables were specified as fixed effects and included plate number (categorical) and array row (continuous). As these models were fitted on a set of unrelated individuals, no adjustment for family relatedness was necessary.

Results from analyses additionally adjusted for trichotomous smoking (coded as 0 for never smokers, 1 for ex-smokers and 2 for current smokers), C-reactive protein (CRP; in mg/L), and extended cell types[11] were also investigated. In the latter, Basophils (Bas), memory B-cells (Bmem), naïve B-cells (Bnv), CD4+ memory T-cells (CD4mem), CD4+ naïve T-cells (CD4nv), CD8+ memory T-cells (CD8mem), CD8+ naïve T-cells (CD8nv), eosinophils (Eos), monocytes (Mono), natural killer (NK) cells, and regulatory T-cells (Treg) were added to the base model above. Neutrophils (Neu) were excluded to avoid collinearity (as proportions for all cells sum to 1).

Lastly, we ran a reverse analysis to further distinguish hsCRP and IL-6 signals. Here, at each of i CpGs for j individuals (n=668), linear models for natural log-transformed CRP (in mg/L) were fitted in R using the `lmFit` function from the Bioconductor `limma` package (see Formula 2).

$$\mathrm{DNAm}_{\beta_j} = \beta_0 + \beta_1 \cdot \ln(CRP)_i + \beta_2 \cdot \mathrm{age}_i + \beta_3 \cdot \mathrm{sexFemale}_i + \beta_4 \cdot \mathrm{CD8T}_i + \beta_5 \cdot \mathrm{CD4T}_i + \beta_6 \cdot \mathrm{NK}_i + \beta_7 \cdot \mathrm{Mono}_i + \beta_8 \cdot \mathrm{plate1}_i + \cdots + \beta_{17} \cdot \mathrm{plate10}_i + \beta_{18} \cdot \mathrm{arrayRow}_i \quad [2]$$

Variables were as described above, and a single sensitivity analysis adjusted for IL-6 levels (in pg/mL) was also performed.

Kooperative Gesundheitsforschung in der Region Augsburg (KORA)

Inflammatory markers Blood samples from KORA F4 participants were drawn after an 8 hour fast and prepared according to the recommendations of the International Committee for Standardization in Hematology[12]. Briefly, blood was drawn into serum gel tubes, gently inverted twice, and then allowed to rest for 30 minutes at room temperature (18-25˚C) to obtain complete coagulation. The sample was then centrifuged for 10 minutes (2750g at 15˚C). Serum was divided into aliquots and kept for a maximum of 6 hours at 4˚C, after which it was frozen at -80˚C until analysis.

Serum concentrations of circulating IL-6 were determined with the Human IL-6 Quantikine HS ELISA kit (R&D Systems, Wiesbaden, Germany). Intra-assay and inter-assay coefficients of variation for IL-6 were 7.2% and 11.8%, respectively[13]. High-sensitivity CRP (hsCRP) was determined in plasma using a high-sensitivity latex-enhanced nephelometric assay on a BN II analyzer (Dade Behring), with intra-assay and inter-assay CVs of 2.7% and 6.3%, respectively[13].

DNA methylation data A random subset of KORA F4 participants (n=1,727) was selected for epigenetic profiling. Following isolation according to standard procedures, genomic DNA (1000 ng) from whole blood was bisulfite converted using the Zymo EZ-96 DNA Methylation Kit (Zymo Research Corp, Irvine, CA, USA). 4 µl from each sample underwent amplification, enzymatic fragmentation, and application to the Infinium HumanMethylation450 BeadChip array (Illumina

Inc, San Diego, CA, USA) according to the manufacturer's protocol. The arrays were fluorescently stained and scanned with the Illumina HiScan SQ scanner.

GenomeStudio with methylation module was used to extract and process the raw image data generated by BeadArray Reader. Initial quality assessment of assay performance was conducted using the Control Dashboard and used control probes present on the array to assess each step in the protocol. For data preprocessing, a common pipeline[14] with default parameter settings was used. Unreliable or outlying values were removed including those based on less than three functional beads or that were indistinguishable from background noise (detection p-value > 0.01) were removed from the analysis.

Methylation proportion at a given cytosine was calculated from the extracted raw methylated (M) and unmethylated (U) probe intensities. This is reported as a beta value, taking values between 0, which represents a completely unmethylated state, and 1, which represents a completely methylated state. Colour bias adjustment based on a smooth quantile normalization method as well as background level correction using negative control probes was performed for each array with the *lumi* R package[15]. Finally, quantile normalization was applied using *wateRmelon*[16] to correct for the InfI/InfII distribution shift of the β values[17].

Model specification At each of i CpGs for j individuals (n=799), linear models for natural log-transformed IL-6 (in pg/mL) were fitted in R using the `lm` function from the `stats` package (see Formula 3).

$$\mathrm{DNAm}_{\beta_j} = \beta_0 + \beta_1 \cdot \ln(IL6)_i + \beta_2 \cdot \mathrm{age}_i + \beta_3 \cdot \mathrm{sexFemale}_i + \beta_4 \cdot \mathrm{CD8T}_i + \beta_5 \cdot \mathrm{CD4T}_i + \beta_6 \cdot \mathrm{NK}_i + \beta_7 \cdot \mathrm{Mono}_i + \beta_8 \cdot \mathrm{controlPC}_i + \cdots + \beta_{37} \cdot \mathrm{controlPC30}_i \quad [3]$$

Age (in years) and sex (coded as 0 for males and 1 for females) were added as continuous and binary fixed effects, respectively. Cell type proportions were predicted from DNA methylation data using the Houseman algorithm[18]. The top 30 control probe PCs were included in all models

as fixed effects to adjust for technical variation. As these models were fitted on a set of unrelated individuals, no adjustment was necessary for family relatedness.

Results from analyses additionally adjusted for trichotomous smoking (coded as 0 for never smokers, 1 for ex-smokers and 2 for current smokers), C-reactive protein (CRP; in mg/L), and extended cell types[11] were also investigated. In the latter, Basophils (Bas), memory B-cells (Bmem), naïve B-cells (Bnv), CD4+ memory T-cells (CD4mem), CD4+ naïve T-cells (CD4nv), CD8+ memory T-cells (CD8mem), CD8+ naïve T-cells (CD8nv), eosinophils (Eos), monocytes (Mono), natural killer (NK) cells, and regulatory T-cells (Treg) were added to the base model above. Neutrophils (Neu) were excluded to avoid collinearity (as proportions for all cells sum to 1).

Lastly, KORA ran a reverse analysis to further distinguish CRP and IL-6 signals. Here, at each of i CpGs for j individuals (n=799), linear models for natural log-transformed CRP (in mg/L) were fitted in R using the `lm` function from the `stats` package (see Formula 4).

$$\mathrm{DNAm}_{\beta_j} = \beta_0 + \beta_1 \cdot \ln(CRP)_i + \beta_2 \cdot \mathrm{age}_i + \beta_3 \cdot \mathrm{sexFemale}_i + \beta_4 \cdot \mathrm{CD8T}_i + \beta_5 \cdot \mathrm{CD4T}_i + \beta_6 \cdot \mathrm{NK}_i + \beta_7 \cdot \mathrm{Mono}_i + \beta_8 \cdot \mathrm{controlPC}_i + \cdots + \beta_{37} \cdot \mathrm{controlPC30}_i \qquad [4]$$

Variables were as described above, and a single sensitivity analysis adjusted for IL-6 levels (in pg/mL) was also performed.

Netherlands Twin Register (NTR)

Inflammatory markers Fasted blood samples were obtained during a home visit, or when preferred, at another address or at work by a trained phlebotomist. Participants were instructed to fast as of 10pm the previous night and to refrain as much as possible from medication use and physical exertion on the day of the home visit and, in the case of smokers, to refrain from smoking one hour prior to the home visit. Using a safety lock butterfly needle, whole blood samples were drawn into EDTA blood tubes. To prevent clotting, tubes were inverted gently 8-10 times immediately after collection.

Samples were then transported in melting ice to the central laboratory in Leiden. Average transport time was 196 minutes and sample processing started as soon as the samples arrived at the lab (full sample processing was completed on average 422 minutes after sample collection). Upon arrival, the EDTA tubes were centrifuged for 20 minutes at 2000x *g* at 4˚C. EDTA plasma was harvested and aliquoted (0.5ml), snap-frozen in dry ice, and stored at -30˚C. Interleukin-6 levels were determined in EDTA plasma with an UltraSensitive ELISA (R&D systems, Minneapolis, USA, Quantikine HS HSTA00C)4. Natural log transformed circulating interleukin-6 levels (microg/L) were used in the EWAS analyses, after excluding observations where transformed levels were not within 3IQR of the nearest quartile. CRP was measured in heparin plasma with the Immulite 1000 CRP assay (Diagnostic Product Corporation, USA).

DNA methylation data Blood sampling procedures have been described in detail previously[6]. DNA methylation was assessed with the Infinium HumanMethylation450 BeadChip Kit (Illumina, San Diego, CA, USA) by the Human Genotyping facility (HugeF) of ErasmusMC, the Netherlands (http://www.glimdna.org/) as part of the Biobank-based Integrative Omics Study (BIOS) consortium. DNA methylation measurements have been described previously[7,8]. Genomic DNA (500ng) from whole blood was bisulfite treated using the Zymo EZ DNA Methylation kit (Zymo Research Corp, Irvine, CA, USA), and 4 μl of bisulfite-converted DNA was measured on the Illumina 450k array following the manufacturer's protocol. A number of sample- and probe-level quality checks and sample identity checks were performed. Quality control and normalization have been described in detail previously[19]. In short, sample-level QC was performed using MethylAid[4]. Probes were set to missing in a sample if they had an intensity value of exactly zero, or a detection p>0.01, or a bead count of less than 3. After these steps, probes that failed based on the above criteria in over 5% of the samples were excluded from all samples (only probes with a success rate ≥ 0.95 were retained). Probes were also excluded from all samples if they mapped to multiple locations in the genome[20], or if they had a single nucleotide polymorphism (SNP) within the CpG site (at the C or G position) irrespective of minor allele frequency in the Dutch population[19]. Only autosomal methylation sites were analyzed in the EWAS. The methylation data were normalized with functional normalization[21]. DNA methylation expressed as β values and any data points further than 3IQR from the nearest quartile were excluded.

Model specification At each of i CpGs for j individuals (n=2,894), generalized estimation equation (GEE) models for natural log-transformed IL-6 (in pg/mL) were fitted in R using the gee function from the gee package (see Formula 5).

$$DNAm_{\beta_j} = \beta_0 + \beta_1 \cdot \ln(IL6)_i + \beta_2 \cdot age_i + \beta_3 \cdot sexFemale_i + \beta_4 \cdot CD8T_i + \beta_5 \cdot CD4T_i + \beta_6 \cdot NK_i + \beta_7 \cdot Mono_i + \beta_8 \cdot plate1_i + \cdots + \beta_{37} \cdot plate30_i + \beta_{18} \cdot arrayRow_i \quad [5]$$

Age (in years) and sex (coded as 0 for males and 1 for females) were added as continuous and binary fixed effects, respectively. Cell type proportions were predicted from DNA methylation data using the IDOL algorithm[10]. Technical variables were specified as fixed effects and included plate number (categorical) and array row (continuous). In the GEE models the link function was specified as Gaussian (for continuous data), with 100 iterations and the 'exchangeable' option to account for the correlation structure within families.

Results from analyses additionally adjusted for trichotomous smoking (coded as 0 for never smokers, 1 for ex-smokers and 2 for current smokers), C-reactive protein (CRP; in mg/L), and extended cell types [11] were also investigated. In the latter, Basophils (Bas), memory B-cells (Bmem), naïve B-cells (Bnv), CD4+ memory T-cells (CD4mem), CD4+ naïve T-cells (CD4nv), CD8+ memory T-cells (CD8mem), CD8+ naïve T-cells (CD8nv), eosinophils (Eos), monocytes (Mono), natural killer (NK) cells, and regulatory T-cells (Treg) were added to the base model above. Neutrophils (Neu) were excluded to avoid collinearity (as proportions for all cells sum to 1).

NTR also ran a reverse analysis to further distinguish CRP and IL-6 signals. Here, at each of i CpGs for j individuals (n=2,894), generalized estimation equation (GEE) models for natural log-transformed CRP (in mg/L) were fitted in R using the gee function from the gee package (see Formula 6). Variables were as described above, and a single sensitivity analysis adjusted for IL-6 levels (in pg/mL) was also performed.

$$DNAm_{\beta_j} = \beta_0 + \beta_1 \cdot \ln(CRP)_i + \beta_2 \cdot age_i + \beta_3 \cdot sexFemale_i + \beta_4 \cdot CD8T_i + \beta_5 \cdot CD4T_i + \beta_6 \cdot NK_i + \beta_7 \cdot Mono_i + \beta_8 \cdot plate1_i + \cdots + \beta_{37} \cdot plate30_i + \beta_{18} \cdot arrayRow_i \quad [6]$$

Quality Control

Results from local EWAS were sent by the respective analysts as *.csv* files with columns for CpG name, association effect size, standard error (SE), nominal p-value, and the number of observations that contributed to this analysis. Cohorts also provided other relevant variables, such as mean DNA methylation at the CpG and information on missingness.

The data were inspected, and rows were removed if based on fewer than 50 observations. We also omitted sex chromosomal CpGs, probes that lay within ENCODE Blacklist regions[22], and those previously flagged as polymorphic, cross-reactive, or unreliable[23] from our analyses. Following these steps, we had data for the base model on 411,665 CpGs in LLS, 412,221 in KORA, and 387,548 in NTR.

Supplementary Figures

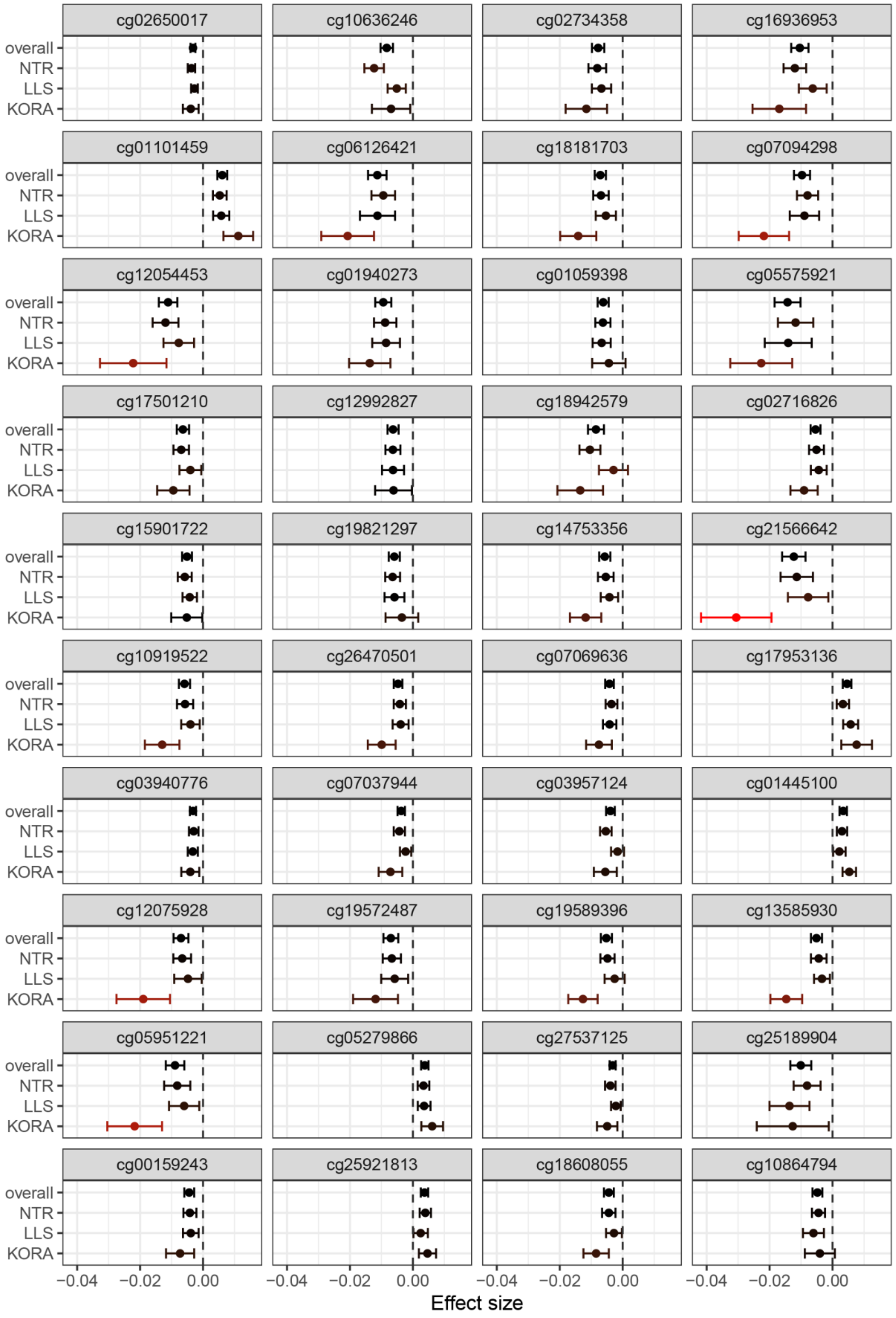

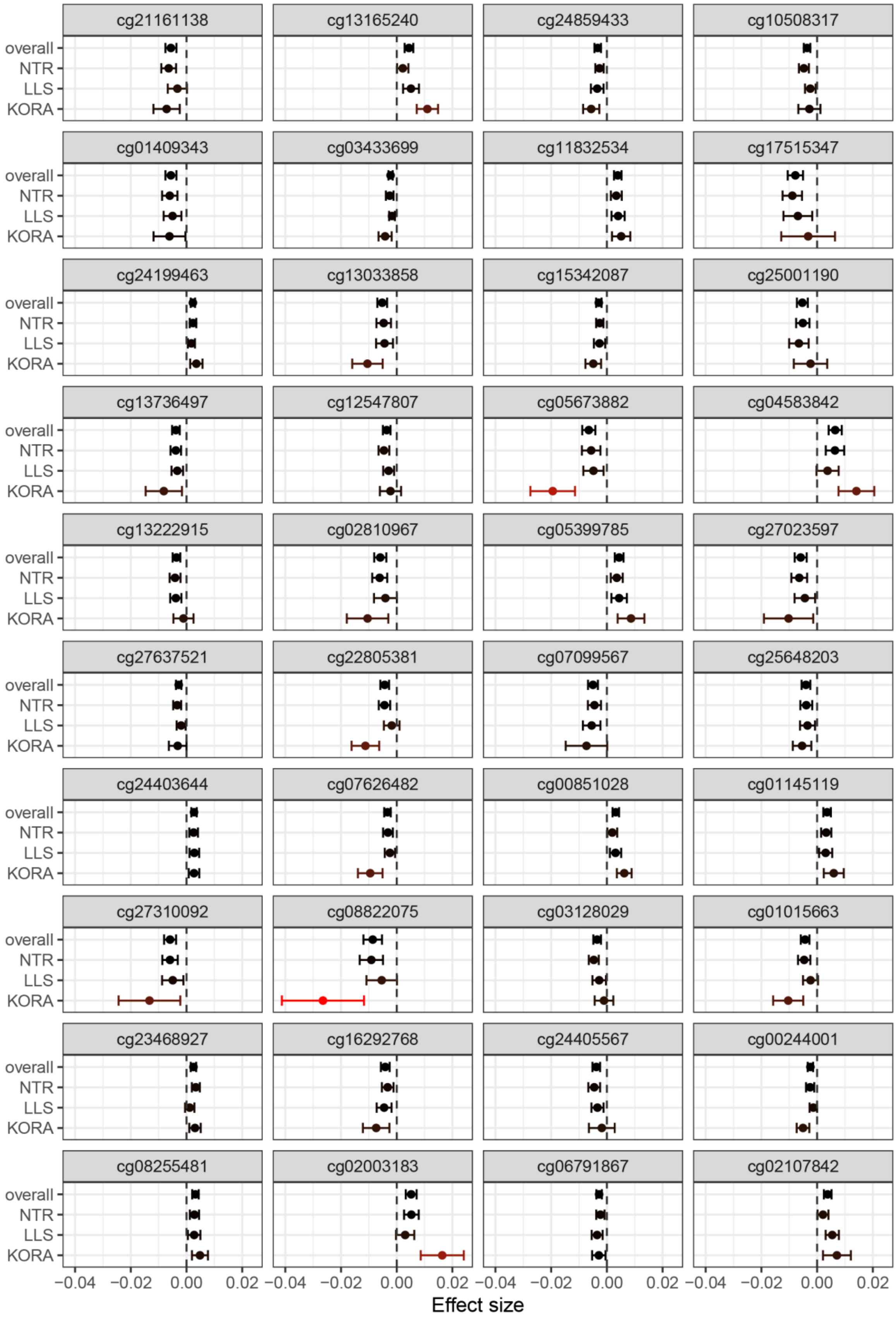
cg21161138
cg13165240
cg24859433
cg10508317
cg01409343
cg03433699
cg11832534
cg17515347
cg24199463
cg13033858
cg15342087
cg25001190
cg13736497
cg12547807
cg05673882
cg04583842
cg13222915
cg02810967
cg05399785
cg27023597
cg27637521
cg22805381
cg07099567
cg25648203
cg24403644
cg07626482
cg00851028
cg01145119
cg27310092
cg08822075
cg03128029
cg01015663
cg23468927
cg16292768
cg24405567
cg00244001
cg08255481
cg02003183
cg06791867
cg02107842
overall
NTR
LLS
KORA
−0.04
−0.02
0.00
0.02
Effect size

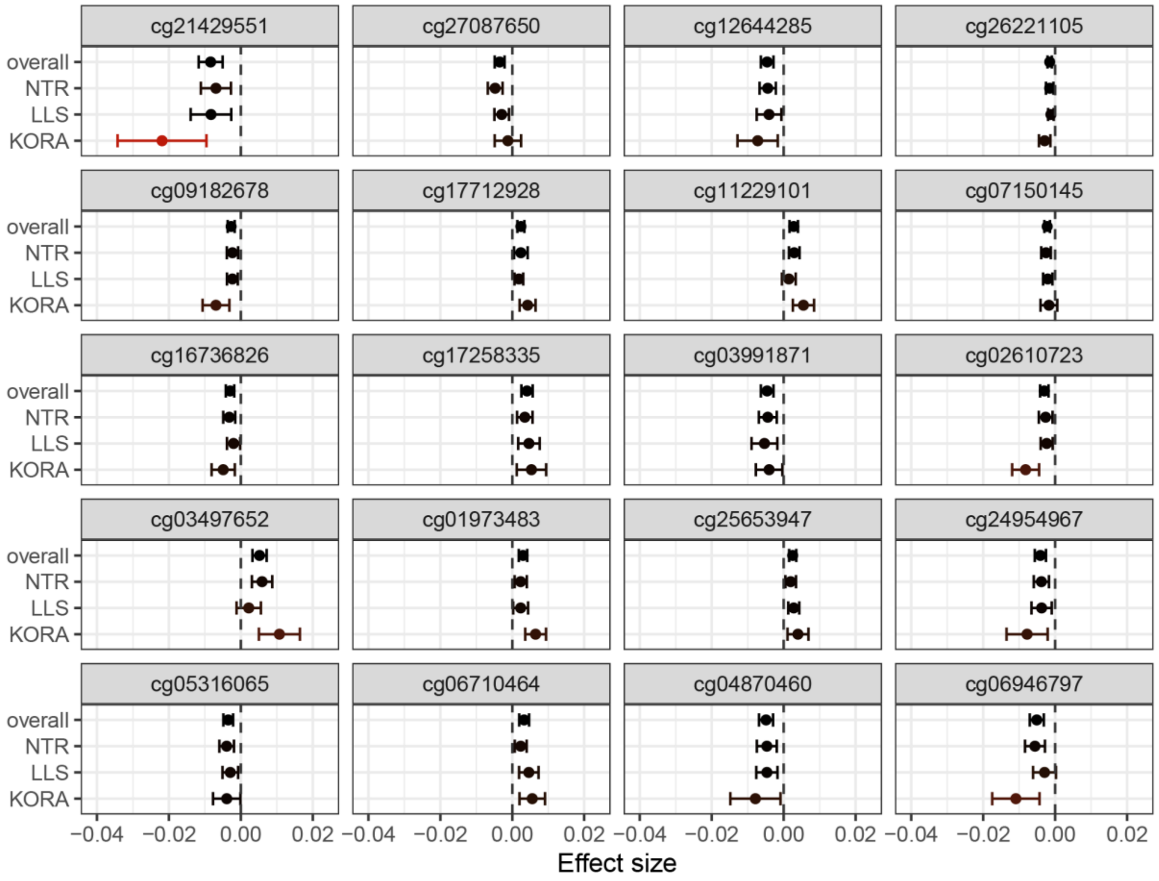

Supplementary Figure 1: Cohort-specific effects for the top 100 CpGs associated with IL-6, ordered by p-value. The effect estimate for each cohort as well as the overall effect is shown alongside the 95% confidence interval, coloured by the absolute difference between the cohort estimate and the overall estimate. A null effect is shown by a grey dashed line.

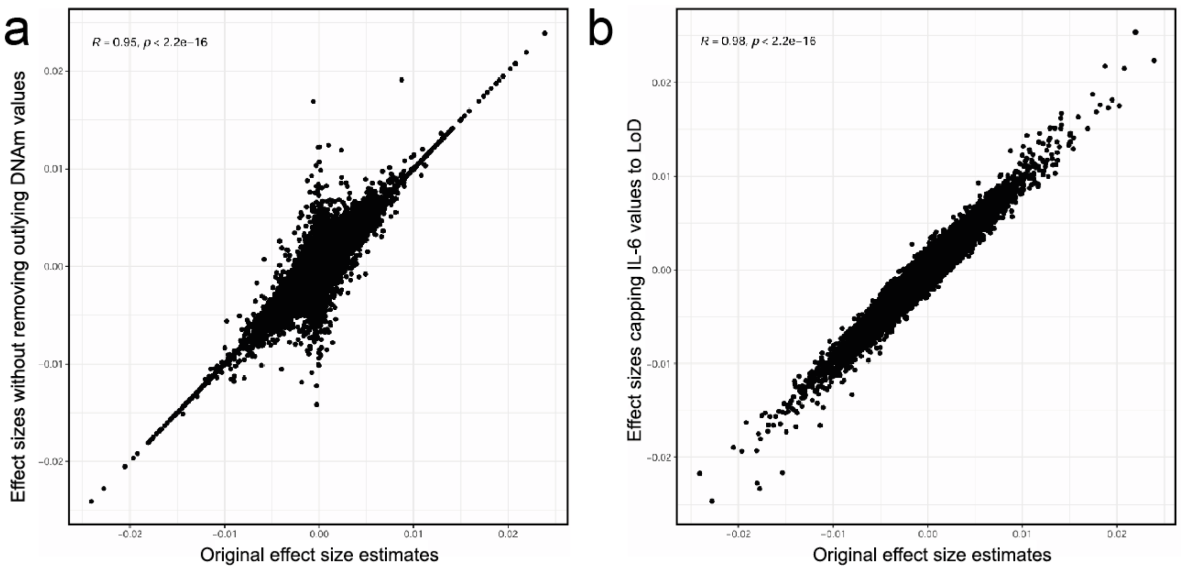

Supplementary Figure 2: Data preprocessing sensitivity analysis results in LLS. (a) Comparing effect sizes with and without removing outlying DNAm values. **(b)** With and without removing IL-6 values below the limit of detection (LoD).

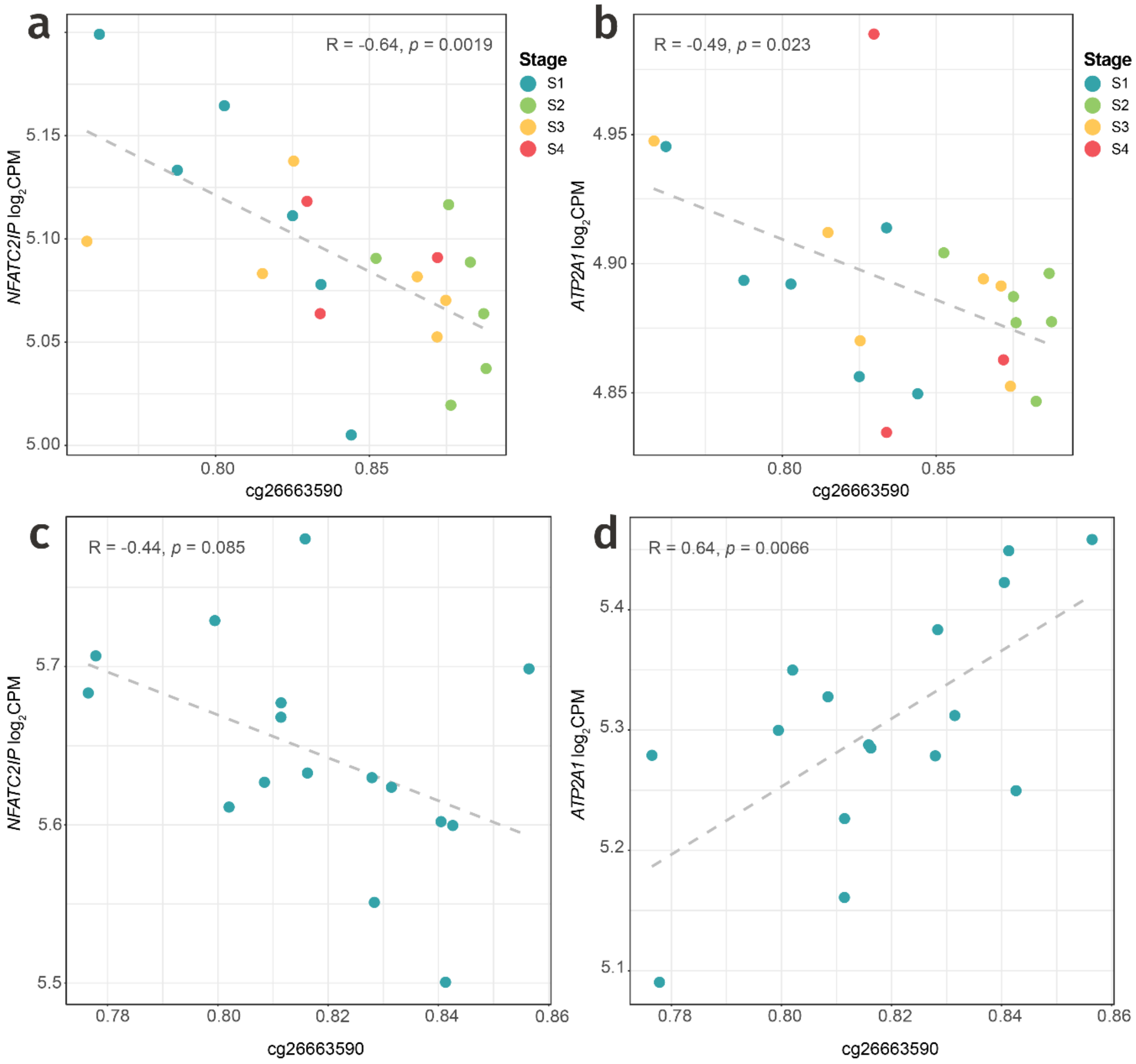

Supplementary Figure 3: eQTM validation in isolated immune cells. (a) Scatterplot of *NFATC2IP* log_2CPM against DNAm at cg26663590 in isolated B-cells at four stages of development, and **(b)** Scatterplot of *ATP2A1* log_2CPM against DNAm in the same cells. **(c)** Scatterplot of *NFATC2IP* log_2CPM against DNAm in CD4+ T-cells, and **(b)** Scatterplot of *ATP2A1* log2CPM against DNAm in the same cells. Pearson correlation coefficients and associated p-values are shown, as well as a line of best fit as a dashed grey line.

References

1. Schoenmaker, M., *et al*. Evidence of genetic enrichment for exceptional survival using a family approach: the Leiden Longevity Study. *European Journal of Human Genetics*, **14**(1), 79-84 (2006).
2. Westendorp, R.G., *et al*. Nonagenarian siblings and their offspring display lower risk of mortality and morbidity than sporadic nonagenarians: The Leiden Longevity Study. *J Am Geriatr Soc*, **57**(9), 1634-7 (2009).
3. Holle, R., *et al*. KORA--a research platform for population based health research. *Gesundheitswesen*, **67 Suppl 1**, S19-25 (2005).
4. Rathmann, W., *et al*. Incidence of Type 2 diabetes in the elderly German population and the effect of clinical and lifestyle risk factors: KORA S4/F4 cohort study. *Diabet Med*, **26**(12), 1212-9 (2009).
5. Meisinger, C., *et al*. Prevalence of undiagnosed diabetes and impaired glucose regulation in 35-59-year-old individuals in Southern Germany: the KORA F4 Study. *Diabet Med*, **27**(3), 360-2 (2010).
6. Willemsen, G., *et al*. The Netherlands Twin Register biobank: a resource for genetic epidemiological studies. *Twin Res Hum Genet*, **13**(3), 231-45 (2010).
7. Bonder, M.J., *et al*. Disease variants alter transcription factor levels and methylation of their binding sites. *Nat Genet*, **49**(1), 131-138 (2017).
8. van Dongen, J., *et al*. Genetic and environmental influences interact with age and sex in shaping the human methylome. *Nat Commun*, **7**, 11115 (2016).
9. van Iterson, M., *et al*. MethylAid: visual and interactive quality control of large Illumina 450k datasets. *Bioinformatics*, **30**(23), 3435-3437 (2014).
10. Koestler, D.C., *et al*. Improving cell mixture deconvolution by identifying optimal DNA methylation libraries (IDOL). *BMC Bioinformatics*, **17**, 120 (2016).
11. Salas, L.A., *et al*. Enhanced cell deconvolution of peripheral blood using DNA methylation for high-resolution immune profiling. *Nature Communications*, **13**(1), 761 (2022).
12. Recommendation for a selected method for the measurement of plasma viscosity. International Committee for Standardization in Haematology. *J Clin Pathol*, **37**(10), 1147-52 (1984).

13. Herder, C., *et al*. Association of subclinical inflammation with polyneuropathy in the older population: KORA F4 study. *Diabetes Care*, **36**(11), 3663-70, (2013).
14. Touleimat, N. and J. Tost, Complete pipeline for Infinium(®) Human Methylation 450K BeadChip data processing using subset quantile normalization for accurate DNA methylation estimation. *Epigenomics*, **4**(3), 325-41 (2012).
15. Du, P., W.A. Kibbe, and S.M. Lin, lumi: a pipeline for processing Illumina microarray. *Bioinformatics*, **24**(13), 1547-8 (2008).
16. Pidsley, R., *et al*. A data-driven approach to preprocessing Illumina 450K methylation array data. *BMC Genomics*, **14**, 293 (2013).
17. Teschendorff, A.E., *et al*. A beta-mixture quantile normalization method for correcting probe design bias in Illumina Infinium 450 k DNA methylation data. *Bioinformatics*, **29**(2), 189-96 (2013).
18. Houseman, E.A., *et al*. DNA methylation arrays as surrogate measures of cell mixture distribution. *BMC Bioinformatics*, **13**(1), 86 (2012).
19. Francioli, L.C., *et al*. Whole-genome sequence variation, population structure and demographic history of the Dutch population. *Nature Genetics*, **46**(8), 818-825 (2014).
20. Chen, Y.A., *et al*. Discovery of cross-reactive probes and polymorphic CpGs in the Illumina Infinium HumanMethylation450 microarray. *Epigenetics*, **8**(2), 203-9 (2013).
21. Fortin, J.P., *et al*. Functional normalization of 450k methylation array data improves replication in large cancer studies. *Genome Biol*, **15**(12), 503 (2014).
22. Amemiya, H.M., A. Kundaje, and A.P. Boyle, The ENCODE Blacklist: Identification of Problematic Regions of the Genome. *Scientific Reports*, **9**(1), 9354 (2019).
23. Zhou, W., P.W. Laird, and H. Shen, Comprehensive characterization, annotation and innovative use of Infinium DNA methylation BeadChip probes. *Nucleic Acids Research*, **45**(4), e22 (2016).
