# Supplementary material for: Epigenome-wide association study of circulating interleukin-6 connects DNA methylation to immunometabolic and inflammatory health
Source: Commun Biol. 2026 Feb 4;9:242. doi: 10.1038/s42003-026-09520-2 (PMC12905258; doi:10.1038/s42003-026-09520-2)
Supplement: Supplementary file 3 — Description of Additional Supplementary Files [file 42003_2026_9520_MOESM3_ESM.pdf]

Description of Additional Supplementary File

File name: Supplementary Data 1

Description: Full summary statistics from the IL-6 EWAS meta-analysis at all tested CpGs

File name: Supplementary Data 2

Description. Sensitivity analyses for the IL-6 associated CpGs, additionally adjusting for 12 cell-types predicted using the IDOLext algorithm and smoking status

File name: Supplementary Data 3

Description: Sensitivity analyses for the IL-6 associated CpGs, additionally adjusting for hsCRP

File name: Supplementary Data 4

Description: Sensitivity analyses for the hsCRP associated CpGs, additionally adjusting for IL-6

File name: Supplementary Data 5

Description: Enrichment of EWAS associations for the IL-6 associated CpGs

File name: Supplementary Data 6

Description: Enrichment of chromatin states for the IL-6 associated CpGs using the PBMC Roadmap reference epigenome (E062)

File name: Supplementary Data 7

Description: Enrichment of chromatin states for the IL-6 associated CpGs using 22 Roadmap reference epigenomes

File name: Supplementary Data 8

Description: Transcription factor binding site enrichment for IL-6 associated CpGs

File name: Supplementary Data 9

Description: eQTM analysis for IL-6 associated CpGs and genes within 100kb

File name: Supplementary Data 10

Description: Colocalisation analysis for IL-6 associated CpGs and genes within 100kb

File name: Supplementary Data 11

Description: Over-representation analysis of genes whose expression is linked to DNAm at IL-6 associated CpGs within 100kb

File name: Supplementary Data 12

Description: TF enrichment analysis for genes whose expression is linked to DNAm at IL-6 associated CpGs within 100kb

File name: Supplementary Data 13

Description: Triangulation of IL-6 on DNAm at IL-6 associated CpGs

File name: Supplementary Data 14

Description: Triangulation of DNAm at IL-6 associated CpGs on IL-6

File name: Supplementary Data 15

Description: Genes responding in isolated CD4+ T-cells in vitro to IL-6 stimulation

File name: Supplementary Data 16

Description: Mediation analysis of DNAm-mediated effects between IL-6 and inflammatory phenotypes

File name: Supplementary Data 17

Description: Two-sample Mendelian randomisation of the effect of DNAm on IL-6 at IL-6 associated CpGs

File name: Supplementary Data 18

Description: Two-sample Mendelian randomisation of the effect of IL-6 on DNAm at IL-6 associated CpGs

File name: Supplementary Data 19

Description: Two-sample Mendelian randomisation of the effect of DNAm at cg26663590 on inflammatory outcomes

File name: Supplementary Data 20

Description: Two-sample Mendelian randomisation of the effect inflammatory outcomes on DNAm at cg26663590
