# Supplementary material for: Epigenome-wide association study of circulating interleukin-6 connects DNA methylation to immunometabolic and inflammatory health
Source: Commun Biol. 2026 Feb 4;9:242. doi: 10.1038/s42003-026-09520-2 (PMC12905258; doi:10.1038/s42003-026-09520-2)
Supplement: Supplementary file 5 — Reporting summary [file 42003_2026_9520_MOESM5_ESM.pdf]

nature portfolio

Corresponding author(s): Lucy Sinke

Last updated by author(s): 14th December 2025

# Reporting Summary

Nature Portfolio wishes to improve the reproducibility of the work that we publish. This form provides structure for consistency and transparency in reporting. For further information on Nature Portfolio policies, see our Editorial Policies and the Editorial Policy Checklist.

Please do not complete any field with "not applicable" or n/a. Refer to the help text for what text to use if an item is not relevant to your study.
For final submission: please carefully check your responses for accuracy; you will not be able to make changes later.

## Statistics

For all statistical analyses, confirm that the following items are present in the figure legend, table legend, main text, or Methods section.

| n/a | Confirmed | |
|---|---|---|
| ☐ | ☑ | The exact sample size ($n$) for each experimental group/condition, given as a discrete number and unit of measurement |
| ☐ | ☑ | A statement on whether measurements were taken from distinct samples or whether the same sample was measured repeatedly |
| ☐ | ☑ | The statistical test(s) used AND whether they are one- or two-sided<br>*Only common tests should be described solely by name; describe more complex techniques in the Methods section.* |
| ☐ | ☑ | A description of all covariates tested |
| ☐ | ☑ | A description of any assumptions or corrections, such as tests of normality and adjustment for multiple comparisons |
| ☐ | ☑ | A full description of the statistical parameters including central tendency (e.g. means) or other basic estimates (e.g. regression coefficient) AND variation (e.g. standard deviation) or associated estimates of uncertainty (e.g. confidence intervals) |
| ☐ | ☑ | For null hypothesis testing, the test statistic (e.g. $F$, $t$, $r$) with confidence intervals, effect sizes, degrees of freedom and $P$ value noted<br>*Give P values as exact values whenever suitable.* |
| ☐ | ☑ | For Bayesian analysis, information on the choice of priors and Markov chain Monte Carlo settings |
| ☑ | ☐ | For hierarchical and complex designs, identification of the appropriate level for tests and full reporting of outcomes |
| ☐ | ☑ | Estimates of effect sizes (e.g. Cohen's $d$, Pearson's $r$), indicating how they were calculated |

*Our web collection on statistics for biologists contains articles on many of the points above.*

## Software and code

Policy information about availability of computer code

| | |
|---|---|
| Data collection | All code underlying data analysis and collection is available at https://github.com/nebulyra/il6_ewas |
| Data analysis | All code underlying data analysis and collection is available at https://github.com/nebulyra/il6_ewas |

For manuscripts utilizing custom algorithms or software that are central to the research but not yet described in published literature, software must be made available to editors and reviewers. We strongly encourage code deposition in a community repository (e.g. GitHub). See the Nature Portfolio guidelines for submitting code & software for further information.

## Data

Policy information about availability of data

All manuscripts must include a data availability statement. This statement should provide the following information, where applicable:

- Accession codes, unique identifiers, or web links for publicly available datasets
- A description of any restrictions on data availability
- For clinical datasets or third party data, please ensure that the statement adheres to our policy

The authors declare that all data supporting the findings of this study are available within the Supplementary Information.

## Research involving human participants, their data, or biological material

Policy information about studies with human participants or human data. See also policy information about sex, gender (identity/presentation), and sexual orientation and race, ethnicity and racism.

| | |
|---|---|
| Reporting on sex and gender | Analyses are adjusted for sex and sex composition of the participating cohorts is clearly defined. |
| Reporting on race, ethnicity, or other socially relevant groupings | All participating cohorts are European and therefore adjustment for race or ethnicity was not relevant for this study. This limitation is clearly addressed in the Discussion. |
| Population characteristics | Table 1 clearly outlines the population demographics of the participating cohorts. |
| Recruitment | Cohorts were contacted to participate in the study. Those with required data types were recruited. |
| Ethics oversight | Ethics approval for the individual cohorts is describing in the Supplementary Methods. |

Note that full information on the approval of the study protocol must also be provided in the manuscript.

# Field-specific reporting

Please select the one below that is the best fit for your research. If you are not sure, read the appropriate sections before making your selection.

☑ Life sciences ☐ Behavioural & social sciences ☐ Ecological, evolutionary & environmental sciences

For a reference copy of the document with all sections, see nature.com/documents/nr-reporting-summary-flat.pdf

# Life sciences study design

All studies must disclose on these points even when the disclosure is negative.

| | |
|---|---|
| Sample size | 4,361 |
| Data exclusions | CpG estimates were excluded is they were based on fewer than 50 observations, were in ENCODE Blacklist regions, on sex chromosomes, or were flagged previously as unreliable. IL-6 measurements below the limit of detection were excluded in cohort specific analyses, and a sensitivity analysis for this step in LLS was performed and is shown in Supplementary Figures. DNAm values more than 3 IQR from the nearest quartile were also excluded in cohort-specific analyses, and a sensitivity analysis for this step was performed in LLS and is shown in the Supplementary Figures. |
| Replication | No replication was performed in this study. |
| Randomization | No randomisation was performed in this study. |
| Blinding | No blinding was performed in this study. |

# Behavioural & social sciences study design

All studies must disclose on these points even when the disclosure is negative.

| | |
|---|---|
| Study description | NA |
| Research sample | NA |
| Sampling strategy | NA |
| Data collection | NA |
| Timing | NA |
| Data exclusions | NA |
| Non-participation | NA |
| Randomization | NA |

# Ecological, evolutionary & environmental sciences study design

All studies must disclose on these points even when the disclosure is negative.

| | |
|---|---|
| Study description | NA |
| Research sample | NA |
| Sampling strategy | NA |
| Data collection | NA |
| Timing and spatial scale | NA |
| Data exclusions | NA |
| Reproducibility | NA |
| Randomization | NA |
| Blinding | NA |

Did the study involve field work? ☐ Yes ☐ No

## Field work, collection and transport

| | |
|---|---|
| Field conditions | NA |
| Location | NA |
| Access & import/export | NA |
| Disturbance | NA |

# Reporting for specific materials, systems and methods

We require information from authors about some types of materials, experimental systems and methods used in many studies. Here, indicate whether each material, system or method listed is relevant to your study. If you are not sure if a list item applies to your research, read the appropriate section before selecting a response.

## Materials & experimental systems

| n/a | Involved in the study | |
|---|---|---|
| ☑ | ☐ | Antibodies |
| ☑ | ☐ | Eukaryotic cell lines |
| ☑ | ☐ | Palaeontology and archaeology |
| ☑ | ☐ | Animals and other organisms |
| ☑ | ☐ | Clinical data |
| ☑ | ☐ | Dual use research of concern |
| ☑ | ☐ | Plants |

## Methods

| n/a | Involved in the study | |
|---|---|---|
| ☑ | ☐ | ChIP-seq |
| ☑ | ☐ | Flow cytometry |
| ☑ | ☐ | MRI-based neuroimaging |

## Antibodies

| | |
|---|---|
| Antibodies used | NA |
| Validation | NA |

## Eukaryotic cell lines

Policy information about cell lines and Sex and Gender in Research

| | |
|---|---|
| Cell line source(s) | NA |
| Authentication | NA |
| Mycoplasma contamination | NA |
| Commonly misidentified lines (See ICLAC register) | NA |

## Palaeontology and Archaeology

| | |
|---|---|
| Specimen provenance | NA |
| Specimen deposition | NA |
| Dating methods | NA |

☐ Tick this box to confirm that the raw and calibrated dates are available in the paper or in Supplementary Information.

| | |
|---|---|
| Ethics oversight | NA |

Note that full information on the approval of the study protocol must also be provided in the manuscript.

## Animals and other research organisms

Policy information about studies involving animals; ARRIVE guidelines recommended for reporting animal research, and Sex and Gender in Research

| | |
|---|---|
| Laboratory animals | NA |
| Wild animals | NA |
| Reporting on sex | NA |
| Field-collected samples | NA |
| Ethics oversight | NA |

Note that full information on the approval of the study protocol must also be provided in the manuscript.

## Clinical data

Policy information about clinical studies

All manuscripts should comply with the ICMJE guidelines for publication of clinical research and a completed CONSORT checklist must be included with all submissions.

| | |
|---|---|
| Clinical trial registration | NA |
| Study protocol | NA |
| Data collection | NA |
| Outcomes | NA |

## Dual use research of concern

Policy information about dual use research of concern

### Hazards

Could the accidental, deliberate or reckless misuse of agents or technologies generated in the work, or the application of information presented in the manuscript, pose a threat to:

| No | Yes | |
|---|---|---|
| ☑ | ☐ | Public health |
| ☑ | ☐ | National security |
| ☑ | ☐ | Crops and/or livestock |
| ☑ | ☐ | Ecosystems |
| ☑ | ☐ | Any other significant area |

### Experiments of concern

Does the work involve any of these experiments of concern:

| No | Yes | |
|---|---|---|
| ☑ | ☐ | Demonstrate how to render a vaccine ineffective |
| ☑ | ☐ | Confer resistance to therapeutically useful antibiotics or antiviral agents |
| ☑ | ☐ | Enhance the virulence of a pathogen or render a nonpathogen virulent |
| ☑ | ☐ | Increase transmissibility of a pathogen |
| ☑ | ☐ | Alter the host range of a pathogen |
| ☑ | ☐ | Enable evasion of diagnostic/detection modalities |
| ☑ | ☐ | Enable the weaponization of a biological agent or toxin |
| ☑ | ☐ | Any other potentially harmful combination of experiments and agents |

## Plants

Seed stocks: NA

Novel plant genotypes: NA

Authentication: NA

## ChIP-seq

### Data deposition

☐ Confirm that both raw and final processed data have been deposited in a public database such as GEO.

☐ Confirm that you have deposited or provided access to graph files (e.g. BED files) for the called peaks.

Data access links
*May remain private before publication.*: NA

Files in database submission: NA

Genome browser session (e.g. UCSC): NA

### Methodology

Replicates: NA

Sequencing depth: NA

Antibodies: NA

Peak calling parameters: NA

Data quality: NA

Software NA

## Flow Cytometry

### Plots

Confirm that:

☐ The axis labels state the marker and fluorochrome used (e.g. CD4-FITC).

☐ The axis scales are clearly visible. Include numbers along axes only for bottom left plot of group (a 'group' is an analysis of identical markers).

☐ All plots are contour plots with outliers or pseudocolor plots.

☐ A numerical value for number of cells or percentage (with statistics) is provided.

### Methodology

| | |
|---|---|
| Sample preparation | NA |
| Instrument | NA |
| Software | NA |
| Cell population abundance | NA |
| Gating strategy | NA |

☐ Tick this box to confirm that a figure exemplifying the gating strategy is provided in the Supplementary Information.

## Magnetic resonance imaging

### Experimental design

| | |
|---|---|
| Design type | NA |
| Design specifications | NA |
| Behavioral performance measures | NA |

| | |
|---|---|
| Imaging type(s) | NA |
| Field strength | NA |
| Sequence & imaging parameters | NA |
| Area of acquisition | NA |

Diffusion MRI ☐ Used ☐ Not used

### Preprocessing

| | |
|---|---|
| Preprocessing software | NA |
| Normalization | NA |
| Normalization template | NA |
| Noise and artifact removal | NA |
| Volume censoring | NA |

### Statistical modeling & inference

| | |
|---|---|
| Model type and settings | NA |
| Effect(s) tested | NA |

Specify type of analysis: ☐ Whole brain ☐ ROI-based ☐ Both

| | |
|---|---|
| Statistic type for inference<br>(See Eklund et al. 2016) | NA |
| Correction | NA |

## Models & analysis

| n/a | Involved in the study |
|---|---|
| ☑ | ☐ Functional and/or effective connectivity |
| ☑ | ☐ Graph analysis |
| ☑ | ☐ Multivariate modeling or predictive analysis |

| | |
|---|---|
| Functional and/or effective connectivity | NA |
| Graph analysis | NA |
| Multivariate modeling and predictive analysis | NA |

This checklist template is licensed under a Creative Commons Attribution 4.0 International License, which permits use, sharing, adaptation, distribution and reproduction in any medium or format, as long as you give appropriate credit to the original author(s) and the source, provide a link to the Creative Commons license, and indicate if changes were made. The images or other third party material in this article are included in the article's Creative Commons license, unless indicated otherwise in a credit line to the material. If material is not included in the article's Creative Commons license and your intended use is not permitted by statutory regulation or exceeds the permitted use, you will need to obtain permission directly from the copyright holder. To view a copy of this license, visit http://creativecommons.org/licenses/by/4.0/
